# Lie Group Analysis of the Photo-Induced Fluorescence of *Drosophila* Oogenesis with the Asymmetrically Localized Gurken Protein

**DOI:** 10.1371/journal.pone.0065143

**Published:** 2013-06-19

**Authors:** Jen-Cheng Wang, Pei-Yu Wang, Hung-Ing Chen, Kai-Ling Wu, Li-Mei Pai, Tzer-En Nee

**Affiliations:** 1 Graduate Institute of Electro-Optical Engineering and Department of Electronic Engineering, Chang Gung University, Kwei-Shan, Tao-Yuan 333, Taiwan, Republic of China; 2 Graduate Institute of Biomedical Sciences, Chang Gung University, Kwei-Shan, Tao-Yuan 333, Taiwan, Republic of China; 3 Graduate Institute of Biomedical Sciences and Department of Biochemistry, Chang Gung University, Kwei-Shan, Tao-Yuan 333, Taiwan, Republic of China; Technische Universität Dresden, Germany

## Abstract

Lie group analysis of the photo-induced fluorescence of *Drosophila* oogenesis with the asymmetrically localized Gurken protein has been performed systematically to assess the roles of ligand-receptor complexes in follicle cells. The (2×2) matrix representations resulting from the polarized t*issue spectra were employed to characterize the* asymmetrical Gurken distributions. It was found that the fluorescence of the wild-type egg shows the Lie point symmetry *X*_23_ at early stages of oogenesis. However, due to the morphogen regulation by intracellular proteins and extracellular proteins, the fluorescence of the embryogenesis with asymmetrically localized Gurken expansions exhibits specific symmetry features: Lie point symmetry *Z*_1_ and Lie point symmetry *X*_1_. The novel approach developed herein was successfully used to validate that the invariant-theoretical characterizations are consonant with the observed asymmetric fluctuations during early embryological development.

## Introduction

The understanding of how multicelluar organisms regulate and control proliferation, differentiation, and cell survival during embryonic development is one of the biggest challenges facing science [1]. Quantitative characterization of the morphogen gradients is vital for understanding their effects on the determination of cell-fate [2]. Several experimental methods have been proposed and successfully performed for the analysis of biological processes [3], such as cell cycle dynamics [4]–[7], cell differentiation [8]–[10], and cell death [11]–[13].

In the *Drosophila melanogaster*, investigations suggest that Gurken is the transforming growth factor (TGFα)-related morphogen that promotes different levels of epidermal growth factor receptor (Egfr) signaling in follicle cells along the dorsal-ventral axis of fruit fly egg chambers [14]–[16]. The developments of higher organisms are derived from embryos, while the embryo is composed of germ cells. They are divided into asymmetrical differentiation and resulting embryo axis, whereas this mechanism is generated by the regulation of concentration gradient distribution in the cells. This axis which arose in the *Drosophila* oogenesis determines the developmental stages. There are many of them participate in the regulation of signal paths. The determination of the body axis is mainly by a specific developmental stage in the performance of the *Drosophila* oogenesis, called Gurken, which activate the neighboring molecules cell's membrane epidermal growth factor receptor, triggering a downstream result of the molecular mechanism of transmission. In the middle of the *Drosophila* oogenesis, Gurken along the dorsal-ventral axis formation in the oocytes showed the concentration gradient and asymmetric distribution between the dorsal and the ventral, and this dorsal-ventral asymmetric distribution becomes the key point to the dorsal-ventral axis. In recent studies, confocal microscopic images have revealed that an expansion of the Gurken distribution is positively regulated by the Dally-like protein (Dlp) causes, while the extent of the Gurken gradient is negatively regulated by *Drosophila* Casitas B-lineage lymphoma Long form-type (D-CblL) [14]. It has been demonstrated that the distribution of the Gurken morphogen is shaped by the receptor endocytosis and can be influenced by the levels of Dlp expression. Development range, defects in the receptor endocytosis or overexpression of Dlp both lead to an expansion of the Gurken gradient. However, overexpression of D-CblL can lead to a significant reduction in the extent of the Gurken gradient due to enhanced capture and subsequent endocytosis of Gurken-Egfr complexes [16]. Apparently, as far as the experimental embryology is concerned, the findings suggest that the morphogens in the follicle cells is regulated by the extracellular and intracellular proteins.

However, to advance our understanding in classical developmental biology, in the past few years, interdisciplinary research and collaboration across a wide array of professional disciplines has become the trend. For example, recently, based on several simple combinatorial codes defined in computer graphics, the gene expression patterns seen in follicle cells have been successfully modeled by the operations of union, difference, intersection, and addition [17]. Biophysical modeling, dimensional analysis, and the quantitative characterization of the transcriptional response to morphogens against a number of genetic backgrounds are considered. Quantitative characterization of the shape of the Gurken gradient has been also been achieved by utilizing the value of the Thiele modulus [2]. In 1952, Alan Turing proposed simple but subtle reaction–diffusion equations, i.e., an eleven-dimensional Lie algebra, to formulate the spatial homogeneity of biochemical reactions in developmental biology [18]. It is well-known that there are four main types of symmetry in the reaction-diffusion equation, namely translation, rotation, inversion, and dilation [19]–[21]. Essentially, since biological systems are also physical systems, we believe that the complexity and integration of living organisms can be mathematically formulated in the context of the symmetry principle originally derived from abstract algebra. The major reason that mathematical modeling is required in biology that rather simple interactions can have interesting consequences that cannot be prognostic for presentment based on biological involvement alone. By careful inspection of the well-established Lie algebra commutation relations, it could be found that these change in the intrinsic symmetry properties of the wild-type egg chambers are related to the shape of the Gurken gradient and subject to the influence of protein regulations during the development processes.

However, very little work has been devoted to the operation of invariant theory or continuous group theory for the understanding of morphogen gradients or even in biology-related research due to the difficulty of the abstract perspective, i.e., Lie group theory, [19]–[22]. While it is true that most multicellular organisms are considered in their entirety to possess unsymmetry, many bio-molecules and tissues do have intrinsic local symmetry. It is believed that the continuous-group approach will be a novel tool to obtain important insights into what proteins with activity intrinsically break the symmetry types, resulting in reshaping of the morphogen gradient. By means of the reaction–diffusion equations, it is corroborated the significance of the Lie group analysis on the examinations of the correlation between the corresponding operations of its non-symmetrical shape and the observed pattern formation of Gurken distribution. In this work, we focus on the Gurken gradient distribution and the composition of the non-symmetrical pattern in the developmental stages of *Drosophila* oogenesis to produce a series of researches. Expecting to use these research methods to lead into the bio-molecular, and hope that we can have a deeper understanding to the asymmetric pattern formation of distribution during the process of biological development.

In the present work, *Drosophila* egg chambers are prepared with intracellular proteins and extracellular proteins, respectively. The asymmetrically localized Gurken gradients in the stage 10A egg chambers are then observed by confocal microscopy. The (2×2) matrix representations resulting from the polarized photoinduced flourescence measurement can be employed to assess the roles of the ligand-receptor complexes in the follicle cell. We confirm that the invariant analysis provides a new measure of the photoinduced tissue spectra in the developmental stages of *Drosophila* oogenesis. Further corroboration of the correlation between the admitting Lie symmetry operations and the observed Gurken localization are carried out by means of the reaction-diffusion equation in a prolate spheroidal coordinate system. The findings concerning the invariant effects of the symmetry law on the Gurken protein expansions could provide a new paradigm for understanding how the group-theory approach governs the fluctuating asymmetry and the developmental stability in a wide variety of organisms.

## Materials and Methods

Fly crosses were grown using the standard procedures at 25°C. The following stocks were used: *D-cbl^F165^*/*TM3* [23], *GR1-Gal4* and *OreR*. The *GR1-Gal4* and *OreR* were found in the Bloomington stock center. The ovaries were dissected in a 1 XPBS buffer and fixed with 200 µl 4% paraformaldehyde in a PBS plus 600 µl heptane and 0.25% NP-40 for 20 minutes at room temperature. For antibody staining, the ovaries were blocked in the PBST plus 1% BSA for one hour. After blocking, the anti-Gurken (1∶50, hydridoma bank (DSHB)) was added and incubated at 4°C overnight. To visualize the actin, 10 units of phalloidin (Molecular Probes) were added for 30 minutes. After incubation with the fluorescent secondary antibody (1∶1000, Molecular Probes) for one hour and then washed several times, the ovaries were mounted in glycerol and examined by the confocal microscopy.

Confocal microspectroscopy is the combination of confocal microscopy with traditional fluorescence spectroscopy. Spectra were collected using a BX41 Olympus optical microscope, equipped with one air-cooled charge-coupled device (CCD) camera which was used for standard point spectroscopy. The attached microscope was a BX41 Olympus optical microscope equipped with four objectives (×100/0.9 NA, ×50/0.75 NA, ×10/0.25 NA, and ×50/0.5 NA) and integrated with a spectrophotometer and a trinocular viewer that could accommodate a video camera allowing direct viewing of the sample. This allowed the collection of photoinduced spectra from different points on the sample, with a spatial resolution of a few micrometers. The spectrometer was an iHR550 model from HORIBA Jobin Yvon, with a focal length of 0.55 m, a Czerny-Turner design, stray light 1×10^−5^ equipped with 1200 gr/mm and 2400 gr/mm gratings. The Rayleigh cutoff was less than 100 cm^−1^ with a notch filter. The thermoelectric cooled CCD (Symphony, 1024×256, Front Illuminated Open Electrode, working temperature –130°C) covered a wide spectral range (200–900 nm). Sample excitation was achieved using an argon ion laser (CVI Melles Griot, New Mexico, U.S.A.) emitting at 488 nm and 25 mW. Calibration of the wavelength axis was achieved by recording the spectral luminescence of silicon (one accumulation, 10 s) for both static and extended modes. If necessary, offset correction was performed to ensure that the position of the silicon band was at 520.50±0.10 cm^−1^. Different sets of polarization inputs and outputs used in the polarized spectral measurements would facilitate the characterization of the tissue spectra by the group-theoretical approach. The polarization configurations of polarized photoinduced spectra for the *Drosophila* oogenesis has the 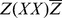, 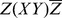, 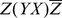, and 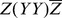 scattering configurations. The input and output polarizations were selected with a half-wave-plate and a polarizer, respectively.

The tissue optical characteristics can be represented by a transformation, i.e.,
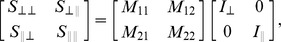
where 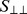, 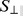, 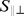, and 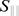 are the scattering luminescent intensities measured with a vertical linear analyzer and a horizontal linear analyzer, respectively; 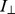 and 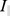 are the incident intensities polarized by a vertical linear polarizer and a horizontal linear polarizer, respectively; and 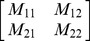 is a cell structure-dependent (2×2) matrix constructed from different polarizer and analyzer measurements. The specific matrix examined is poised to provide many interesting insights into developmental cell features underlying the protein family.

## Results and Discussion

**Figure 1:**
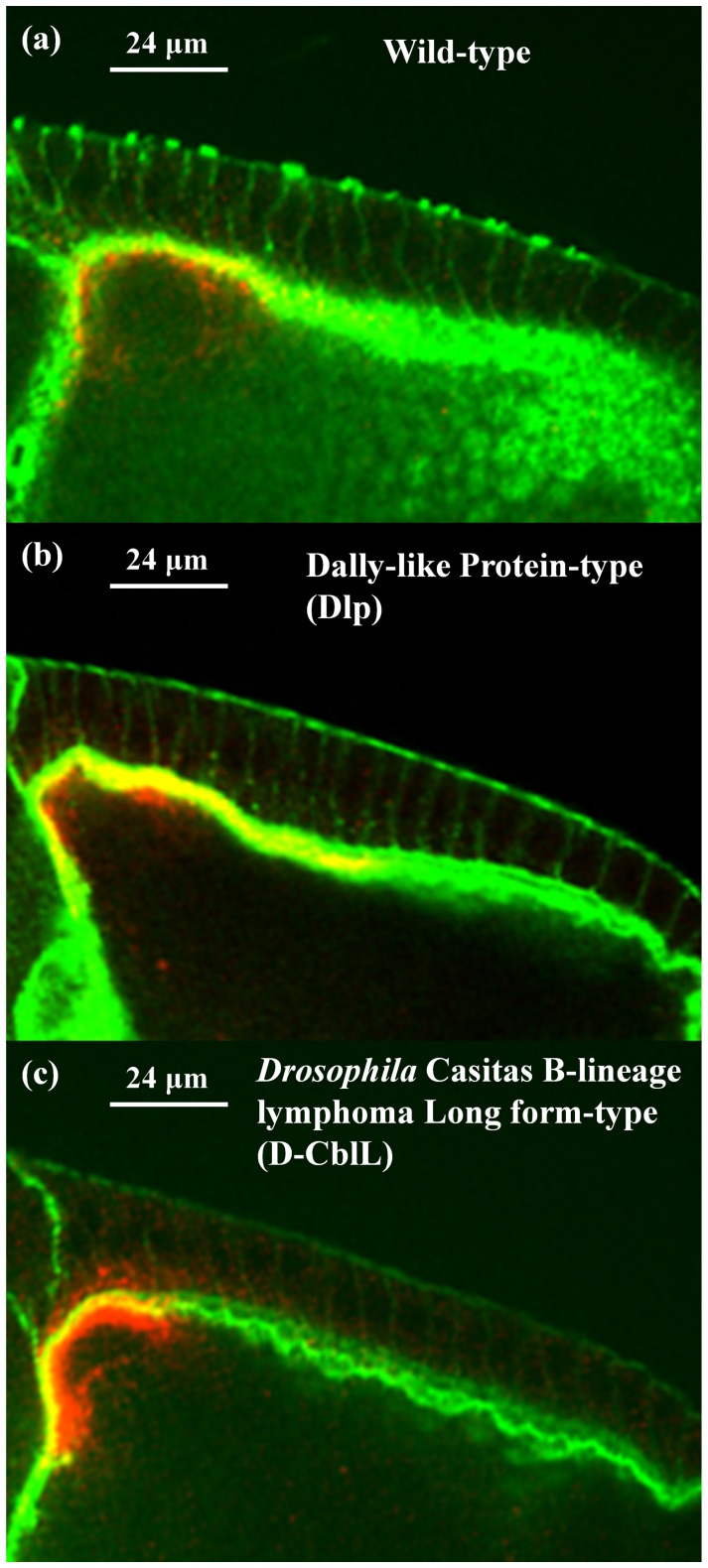
The partial distribution of the Gurken gradient in stage 10A egg chambers for (a) wild-type, (b) overexpressing Dlp, and (c) overexpressing D-CblL. The scale bars both are 24 µm in Figs. 1 (a), (b) and (c).

Confocal microscopy was utilized to show the distribution and spatial resolution of asymmetrically localized Gurken proteins in fluorescently labeled *Drosophila* egg chambers. The green stain, as illustrated in Fig. 1, indicates the partial distribution of the ligand Gurken gradient in the stage 10A egg chambers for (a) wild-type, (b) overexpressing Dlp, and (c) overexpressing D-CblL. Compared with the wild-type fly eggs, it is clear that the over-expression of Dlp leads to an increase in the distance which the Gurken travels, while the over-expression of D-CblL yields a decrease in the shot-range target gene. These observations can be attributed to morphogen regulation by the intracellular and extracellular proteins in the follicle cells of egg chambers, respectively, as noted in our previous report [24].

**Figure 2:**
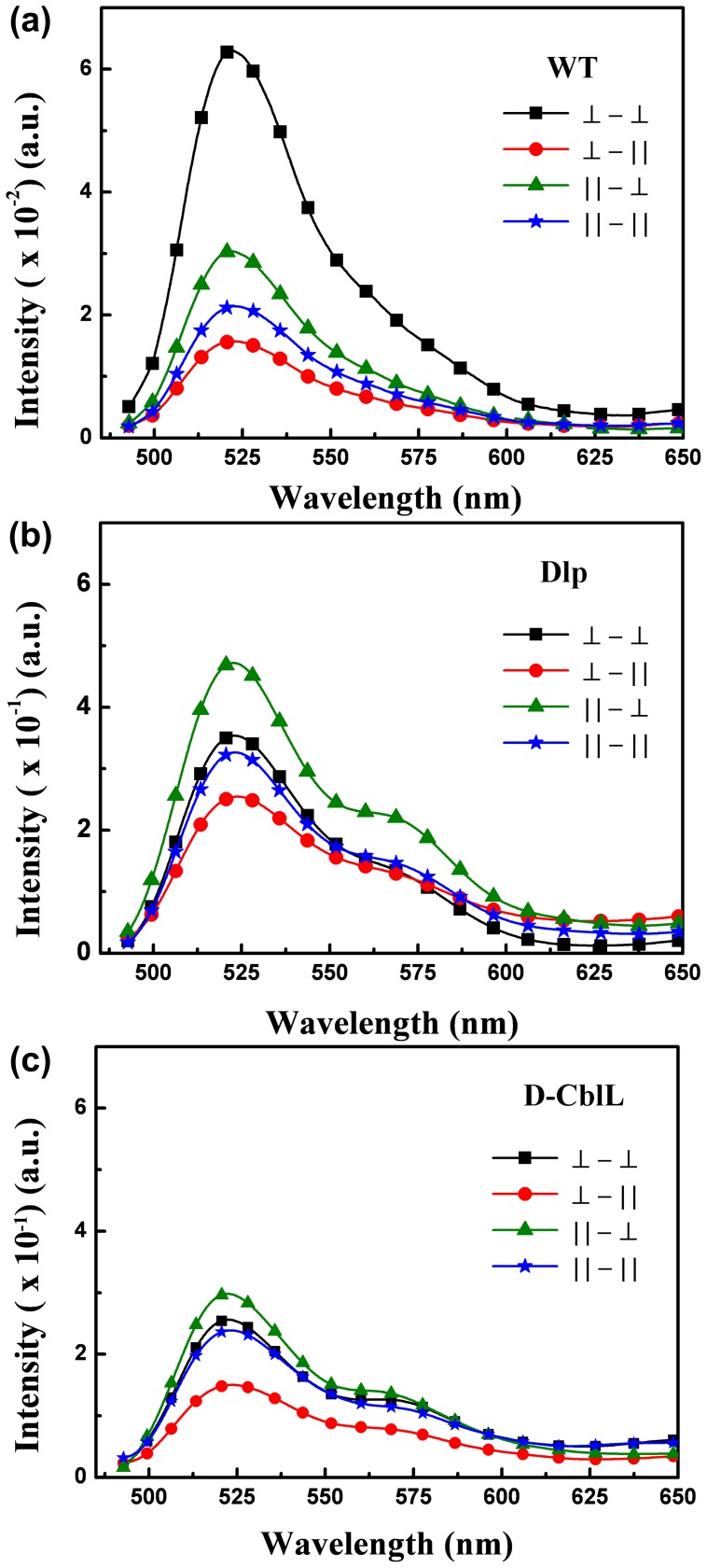
The polarized photo-induced fluorescence spectra showing the global distribution of the Gurken gradient in stage 10A egg chambers for (a) wild-type, (b) overexpressing Dlp, and (c) overexpressing D-CblL after linear background subtraction. The experimental data were contingent on linear polarization by vertical and horizontal polarizers.

To elaborate further on these observations, a systematic study of the scattering luminescence intensity as a function of the incident light polarization was carried out. With the implementation of the four scattering configurations, i.e., 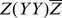, 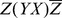, 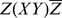, and 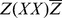, as mentioned above, Figure 2 shows the polarized photoinduced spectra of the global distribution of the Gurken gradient in (a) wild-type, (b) overexpressing Dlp, and (c) overexpressing D-CblL eggs after linear background subtraction. All the probed intensities are normalized relative to the unpolarized forward scattering light for comparison of the data collected by the different optical measurements. Since there is essentially no difference between the spectra of the fluorescent stained and nonstained samples, spectral excitation due to antibody staining can be ruled out in this study. There are two main broadband protein-related emissions observed around 524 nm and 565 nm in the red-green spectral range at 300 K. The explanation for the dependence of these anisotropic endogenous fluorophores on the developmental process is not yet completely understood [25], [26]. Future work shoud be carried out to investigate the significance of spectral analysis combined with structural findings. Accordingly, as mentioned above, the matrix representatives extracted from the normalized fluorescent amplitudes for the wild-type egg *X_WT_*, overexpressing Dlp egg *X_Dlp_*, and overexpressing D-CblL egg *X_D-CblL_* are
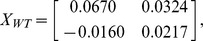


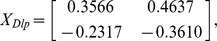
and
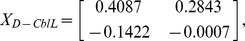
respectively.

Although a cursory glance at these matrices might suggest that the fluorescence emissions are random, at closer inspection symmetry arguments may be very fruitfully applied to reveal the characteristic regularity and simplicity in follicle cells. Remarkably, the commutative multiplication relations between *X_WT_*, *X_Dlp_*, and *X_D-CblL_* can be expressed by
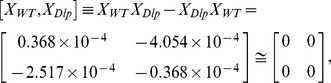


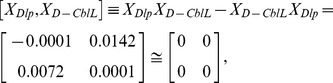
and
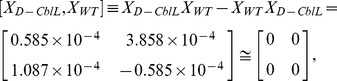
respectively. The connections examined are expected to provide important clues regarding not only the bioluminescent properties of oogenesis, but also the symmetry-related characteristics in the developmental stages.

An eleven-dimensional Lie algebra is carried out based on the prolongation structure of the reaction-diffusion equations describing the morphogen expansion, as proposed by Alan Turing proposed in 1952 [18]. It is well-known that there are four main types of symmetry in the reaction-diffusion equation, namely translation, rotation, inversion, and dilation [19]–[21]. Careful inspection of the well-established Lie algebra commutation relations suggests that these three morphogen-dependent matrices are assigned as follows: point symmetry *X*_23_, point symmetry *X*_1_, and point symmetry *Z*_1_ for wild-type eggs, overexpressing Dlp eggs, and overexpressing D-CblL eggs, respectively [19]–[21]. The change in the intrinsic symmetry properties of the wild-type egg chambers is subject to the influence of protein regulations during the development processes. As a group-theory approach for Gurken redistribution, our data suggest that overexpression of Dlp causes a break in the symmetry from point symmetry *X*_23_ to point symmetry *X*_1_, while overexpression of D-CblL changes point symmetry *X*_23_ to point symmetry *Z*_1_.

**Figure 3:**
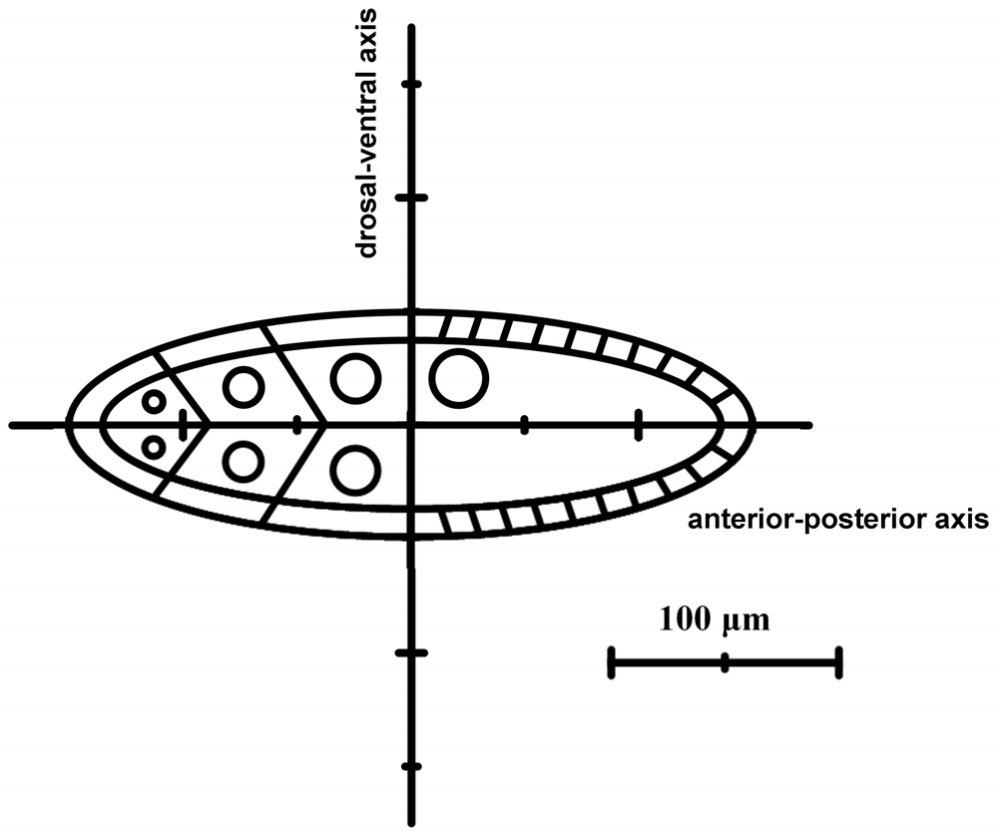
A schematic diagram of the stage 10A *Drosophila* oogenesis chambers with the anterior-posterior-axis and dorsal-ventral axis. The scale bar is 100 µm.

Interestingly, on further assessment of the relevance between the corresponding continuous symmetry operations and the observed Gurken localization, we carry out a Lie group analysis of the reaction-diffusion equations. Since a stage 10A fruit fly egg is approximately a prolate spheroid, the Gurken distributions can be easily described with a two dimensional coordinate system appropriate to the geometry of the oocytes observed. A prolate spheroidal coordinate plane can be created by a horizontal line called the anterior-posterior-axis and a vertical line called the dorsal-ventral axis, as shown in Fig. 3. The conversion relations from prolate spheroidal 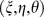 to Cartesian coordinates (*x*, *y*, *z*) are given by 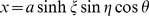, 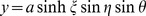, and 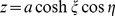, where 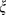 is a nonnegative real number and 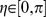. The azimuthal angle 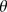 belongs to the interval [0,2π) [27].

Generally, a morphogenesis model describing pattern formation must involve nonlinear coupled reaction-diffusion equations. However, the governing equations regarding to the mechanism of Gurken localization can be completely expanded by the eleven Lie symmetry vector fields [20]–[22]. According to the polarized photoinduced spectra of *Drosophila* oogenesis we have obtained in the present work, the three symmetry operations of *X*_1_, *X*_23_, and *Z*_1_ are of major concern, i.e.,
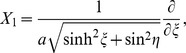


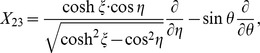
and
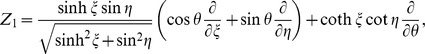
where *X*_1_, *X*_23_, and *Z*_1_ are the translation vector field, the hyperbolic rotation vector field, and the dilation vector field, respectively. Acting on the reaction-diffusion equation with these three infinitesimal generators, the length of the anterior-posterior axis and dorsal-ventral axis of *Drosophila* oogenesis are 320 and 145 µm, respectively; the thickness of the follicle cells is 10 µm. Under a steady state, the boundary value of the oocyte is equal to zero at the two sides of follicle cells.

**Figure 4:**
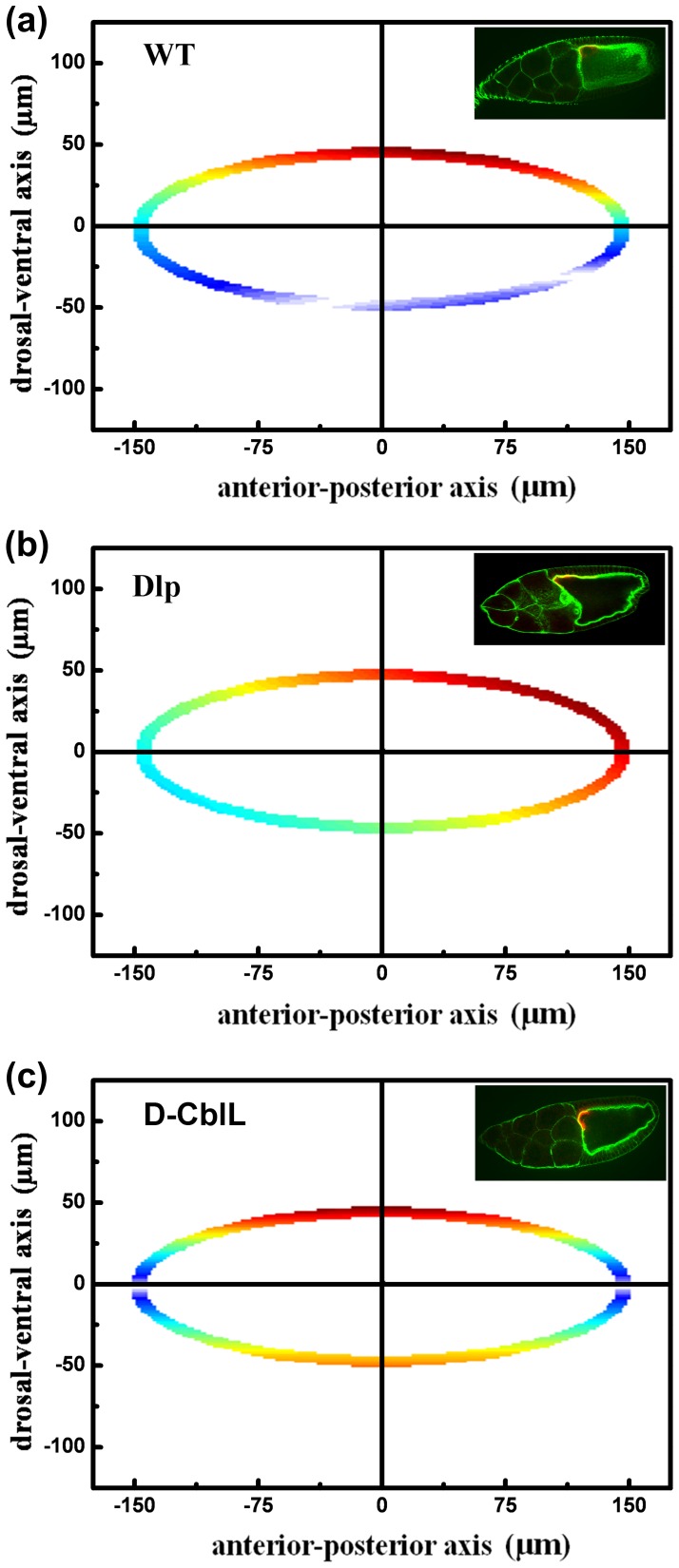
The spatial distribution of the morphogen Gurken gradient in (a) the stage 10A wild-type egg, (b) overexpressing Dlp egg, and (c) overexpressing D-CblL egg chambers which are possessed of (a) *X*_23_ type, (b) *X*_1_ type, and (c) *Z*_1_ type symmetric operators, respectively. The insets show the schematic global distribution of the Gurken gradient in the stage 10A wild-type eggs, overexpressing Dlp eggs, and overexpressing D-CblL eggs, respectively.

Figs. 4 (a), (b) and (c) show the spatial distributions of the morphogen in wild-type eggs, overexpressing Dlp eggs, and overexpressing D-CblL eggs, respectively. Apparently, the results formulated from the infinitesimal symmetry group are consonant with the asymmetric Gurken distributions observed in Fig. 1. The Gurken spreads for wild-type eggs, overexpressing Dlp eggs, and overexpressing D-CblL eggs exhibit rotation transformations, translation transformations, and dilation transformation, respectively, reflecting the intrinsic symmetry of the developmentally regulated expression. Compared with the corresponding polarized bio-fluorescence observations, the pattern formations of the Gurken localizations fully corroborate the point-symmetry assignment for the three samples. Developmentally, the gene expression patterns in the follicle cells are regulated by extracellular proteins or intracellular proteins. Bioluminescently, the polarized tissue spectra of the oogenesis depend on the asymmetrically localized Gurken proteins. Mathematically, however, these specific ligand-receptor complexes subtly break the symmetric features shown in the wild-type eggs reforming to those shown in the Dlp eggs and D-CblL eggs. Consistent with the abstract algebraic results regarding morphogenesis is the fact that both the observed changes in the Gurken gradient and the photoinduced spectra are governed by the principle of symmetry. Implicit in the proposed invariant-theoretical descriptions is the concern that interdisciplinary research and collaboration are permitting a more comprehensive understanding of fluctuating asymmetry and developmental stability in a wide variety of organisms.

## Conclusions

In summary, a Lie group study of the photo-induced fluorescence of *Drosophila* oogenesis with the asymmetrically localized Gurken protein has been carried out. The (2×2) matrix representations extracted from the polarized micro-spectra of morphogen gradient profiles were employed to assess the roles of the ligand-receptor complexes in follicle cells. It was found that the expansions in the Gurken distribution caused by the Dlp and D-CblL reveal the Lie point symmetry *X*_1_ and Lie point symmetry *Z*_1_, respectively, while that of the wild-type egg shows the Lie point symmetry *X*_23_. The convincing correlation between the corresponding continuous symmetry operations and the observed Gurken localization corroborates the significance of the Lie group analysis by means of the reaction-diffusion equation in a prolate spheroidal coordinate system. These findings not only reveal the bioluminescent properties of oogenesis but also confirm the symmetry-related characteristics in the developmental stages.
